# Levosimendan Protects against Doxorubicin-Induced Cardiotoxicity by Regulating the PTEN/Akt Pathway

**DOI:** 10.1155/2020/8593617

**Published:** 2020-06-07

**Authors:** Ling-Li Li, Li Wei, Ning Zhang, Wen-Ying Wei, Can Hu, Wei Deng, Qi-Zhu Tang

**Affiliations:** ^1^Department of Cardiology, Renmin Hospital of Wuhan University, Wuhan 430060, China; ^2^Hubei Key Laboratory of Metabolic and Chronic Diseases, Wuhan 430060, China; ^3^Department of Pediatrics, Renmin Hospital of Wuhan University, Wuhan 430060, China; ^4^Department of Cardiology, The Fifth Affiliated Hospital of Xinjiang Medical University, Ürümqi, China

## Abstract

**Background and Aims:**

Myocyte apoptosis plays a critical role in the development of doxorubicin- (DOX-) induced cardiotoxicity. In addition to its cardiotonic effect, laboratory evidence indicates that levosimendan can inhibit apoptosis, but its role in DOX-induced cardiac injury remains unclear. Therefore, the present study is aimed at exploring whether levosimendan could attenuate DOX-induced cardiotoxicity.

**Methods:**

Levosimendan (1 mg/kg) was administered to mice through oral gavage once daily for 4 weeks, and the mice were also subjected to an intraperitoneal injection of DOX (5 mg/kg) or saline, once a week for 4 weeks, to create a chronic model of DOX-induced cardiotoxicity. A morphological examination and biochemical analysis were used to evaluate the effects of levosimendan. H9C2 cells were used to verify the protective role of levosimendan in vitro. And an Akt inhibitor was utilized to verify the cardioprotection of levosimendan.

**Results:**

Levosimendan reduced the cardiac dysfunction and attenuated the myocardial apoptosis induced by DOX in vivo and in vitro. Levosimendan also inhibited the activation of phosphatase and tensin homolog (PTEN) and upregulated P-Akt expression both in vivo and in vitro. And inhibition of Akt abolished the cardioprotection of levosimendan in vitro.

**Conclusion:**

Levosimendan may protect against DOX-induced cardiotoxicity via modulation of the PTEN/Akt signaling pathway.

## 1. Introduction

In the past few decades, cancer mortality rates have declined, and the side effects caused by anticancer chemotherapeutic agents have become increasingly prominent. Doxorubicin (DOX) is a broad-spectrum anthracycline antibiotic used to treat solid and hematogenous malignancies [1–3]. The cumulative and dose-dependent toxicity induced by DOX is harmful to nontumor tissues, and in myocardial tissue, DOX causes irreversible damage, which can lead to dilated cardiomyopathy, greatly limiting its clinical application [4, 5]. Many mechanisms underlying DOX-induced cardiotoxicity have been discovered, including mitochondrial iron accumulation and related redox reactions, the activation of immunological reactions, histamine release, and DNA damage, and emerging research indicates a crucial role for apoptosis [6–8].

Levosimendan, a calcium sensitizer, is known as a promising positive inotropic and vasodilatory agent used in the treatment of acute heart failure and other circumstances where an improvement in hemodynamics is required [9–12]. Levosimendan may exert its protective effects through the modulation of reactive oxygen species formation, adenosine triphosphate-sensitive potassium channel activity, membrane potential, or the release of endothelial nitric oxide synthase-dependent nitric oxide [13–15]. In addition, previous studies have shown that levosimendan protects against oxidative injury in animal models via interference with apoptotic signaling [16]. Numerous studies have shown that Akt plays pivotal roles in protecting cardiac myocytes from damage by inhibiting apoptosis [16–18]. Akt, also named protein kinase B, is a signaling nexus governing cell growth, cell proliferation, and cell survival [18, 19]. Activation of Akt is regulated by upstream phosphatase and tensin homolog (PTEN), which reduces the phosphorylation of Akt and blocks downstream signaling events regulated by Akt [19–21]. Therefore, the aim of the current study was to investigate the protective effect and the mechanism of levosimendan in DOX-induced cardiotoxicity.

## 2. Materials and Methods

All animal experiments were performed in accordance with the Guide for the Care and Use of Laboratory Animals (National Institutes of Health publication number: 85-23, revised 1996) and approved by the Animal Care and Use Committee of Renmin Hospital of Wuhan University.

### 2.1. Reagents

DOX was purchased from Haizheng Pfizer Pharmaceutical Co., Ltd. Levosimendan was obtained from Orion Corporation, Espoo, Finland. The following primary antibodies were purchased from Cell Signaling Technology (Boston, MA, USA): Bcl-2-associated X protein (BAX; 1 : 1000), c-caspase-3 (1 : 1000), PTEN (1 : 1000), P-Akt (1 : 1000), T-Akt (1 : 1000), and glyceraldehyde-3-phosphate dehydrogenase (GAPDH, 1 : 1000), and B-cell lymphoma-2 (Bcl-2) (1 : 1000) was purchased from Abcam. Akt inhibitor (Akt i) was purchased from Sigma-Aldrich (St. Louis, MO, USA). A goat anti-rabbit secondary antibody was purchased from LI-COR Biosciences (Lincoln, USA). The BCA protein assay kit was obtained from Dōjindo Laboratories (Kumamoto, Japan).

### 2.2. Animals and Treatments

All animal care and experimental procedures were approved by the Animal Care and Use Committee of Renmin Hospital of Wuhan University, which is guided by the Guidelines for the Care and Use of Laboratory Animals published by the United States National Institutes of Health (NIH Publication, revised 2011). All the animal treatments and subsequent analysis were performed in a blind fashion for all groups. Male C57BL/6 mice (8 weeks old; body weight: 25.5 ± 2 g) were purchased from the Institute of Laboratory Animal Science, Chinese Academy of Medical Sciences (Beijing, China). The mice were allowed free access to food and water under a 12 h light-dark cycle with controlled temperature (20–25°C) and humidity (50 ± 5%) in the Cardiovascular Research Institute of Wuhan University (Wuhan, China). A total of 40 male C57BL/6 mice were randomly divided into four groups: control group (CON, *n* = 10), control+levosimendan group (CON+L, *n* = 10), doxorubicin group (DOX, *n* = 10), and doxorubicin+levosimendan group (DOX+L, *n* = 10). The mice were injected intraperitoneally with DOX (5 mg/kg, once a week; the total cumulative dose was 20 mg/kg) or the same dose of saline for 4 weeks. Meanwhile, levosimendan (1 mg/kg) or the same volume of saline was administered through oral gavage once daily for 4 weeks. At the endpoint of the treatment, all the mice were anesthetized using 1.5% isoflurane and euthanized by cervical dislocation. The mouse hearts were dissected and weighed to assess heart weight/tibial length (HW/TL) and then snap-frozen in liquid nitrogen for further analysis.

### 2.3. Echocardiography and Hemodynamics

After the mice were anesthetized using isoflurane (1.5%), echocardiography was performed using a MyLab 30CV ultrasound (Esaote SpA, Genoa, Italy) with a 10 MHz linear array ultrasound transducer. The left ventricle (LV) end-systolic diameter (LVESD), LV end-diastolic dimension (LVEDD), LV ejection fraction (EF), and LV fractional shortening (FS) were measured along the short axis of the left ventricle at the level of the papillary muscles.

Hemodynamic variables were analyzed using a Millar catheter transducer (SPR-839; Millar Instruments, Houston, TX). The maximal rate of pressure development (dp/dtmax) and the minimal rate of pressure decay (dp/dtmin) were processed using PVAN data analysis software (Millar, Inc. Houston, TX, USA). All surgeries and analyses were performed in a blinded manner.

### 2.4. Morphological Analysis

The removed hearts were fixed in 10% formalin overnight. The hearts were transversely sectioned into 5 *μ*m slices. Then, the slices were stained using hematoxylin and eosin (HE). The cross-sectional area (CSA) of the cardiomyocytes was examined based on HE-stained sections using a quantitative digital image analysis system (Image-Pro Plus 6.0). More than 100 myocytes in the LV were outlined in each group.

### 2.5. Western Blotting and Quantitative Real-Time PCR

Total protein was extracted from the frozen heart tissues or H9C2 cells using RIPA agent (Invitrogen, Carlsbad, CA, USA). Then, the Pierce BCA Protein Assay Kit (23227, Thermo Scientific, MIT, USA) was used to measure the concentration of total protein. The proteins were separated through 10% dodecyl sulfate, sodium salt-polyacrylamide gel electrophoresis (SDS-PAGE) and then transferred to polyvinylidene fluoride (PVDF) membranes (cat. number IPFL00010; EMD Millipore, Billerica, MA, USA), and primary antibodies were incubated with the blot at 4°C overnight. After reaction with secondary antibodies at 37°C for 1 h, the blots were scanned and analyzed using a two-color infrared imaging system (Odyssey, LICOR Biosciences, NE, USA). Total RNA was isolated using TRIzol and reverse-transcribed to cDNA. A LightCycler 480 SYBR Green Master Mix (cat. number 04896866001; Roche) was used to quantify amplification. The mRNA data were normalized to GAPDH.

### 2.6. Cell Culture

H9C2 cells were obtained from the Cell Bank of the Chinese Academy of Science (Shanghai, China) and were cultured in Dulbecco's modified Eagle's medium (DMEM, GIBCO, C11995), containing 10% fetal bovine serum (GIBCO, 15140). H9C2 cells were seeded in six-well culture plates or on glass slides and incubated in 5% CO_2_ and 95% air at 37°C. The medium was exchanged for serum-free DMEM 48 h later, to starve the cells for 12 h. Subsequently, the cells were pretreated using levosimendan (10 *μ*mol/mL) or phosphate buffer saline (PBS) for 2 h, and then, DOX (1 *μ*mol/L) was added into the medium in the presence or absence of levosimendan for 24 h. Proteins or mRNA were then harvested. Meanwhile, H9C2 cells were pretreated with AKT i (1 *μ*mol/L) for 30 min, as we previously described [22].

### 2.7. Terminal Deoxynucleotidyl Transferase-Mediated Nick End Labeling (TUNEL) Staining

Briefly, terminal deoxynucleotidyl transferase-mediated nick end labeling (TUNEL) staining was performed using a commercial kit (Millipore, Billerica, MA, USA) in accordance with the manufacturer's instructions. The nuclei of the cardiac myocytes were stained using 4′,6-diamidino-2-phenylindole (DAPI). Subsequently, slices of paraffin-embedded tissue or cells were observed and images were captured using an Olympus DX51 fluorescence microscope (Olympus, Japan).

### 2.8. Statistical Analysis

All data are presented as the mean ± SD. A one-way analysis of variance followed by Tukey's post hoc test was used to analyze differences among groups. *P* < 0.05 was considered to indicate statistical significance.

## 3. Results

### 3.1. Levosimendan Improved Cardiac Function in Mice Undergoing DOX Treatment

**Figure 1:**
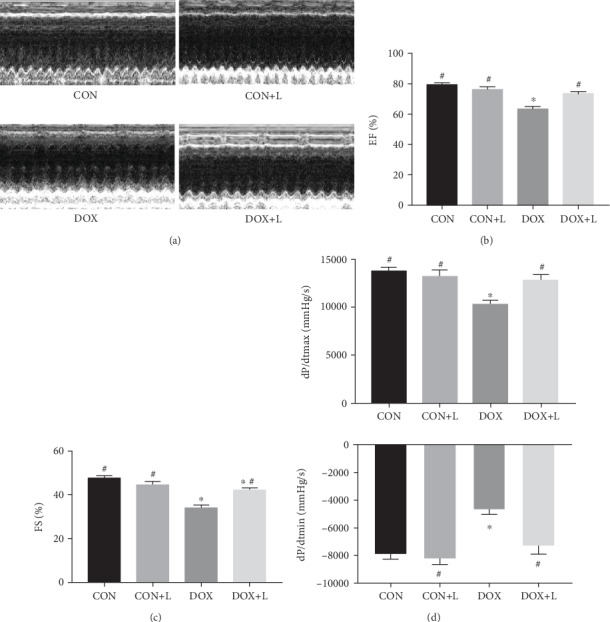
Levosimendan improved cardiac function in mice during DOX treatment. (a) Representative echocardiographic images of each group, including the CON, CON+L, DOX, and DOX+L groups (LVDs: left ventricular internal dimension systole; LVDd: left ventricular internal dimension diastole; IVS: interventricular septal thickness; PW: posterior wall). (b) Left ventricular ejection fraction (EF) of mice with or without treatment with levosimendan 4 weeks after injection of DOX or saline. (c) Fractional shortening (FS) in each group of mice. (d) Hemodynamic analysis of mice including dp/dt max and dp/dt min. ^∗^*P* < 0.05, vs. the CON group; ^#^*P* < 0.05, vs. the DOX group.

After 4 weeks, all 40 mice survived. To determine the effect of levosimendan on cardiac dysfunction, the wall thickness, chamber diameter, and LV function were measured. As shown in Figure 1(a), the wall thickness of the DOX group was thinner and the LVEDD was larger than that of the CON and CON+L groups, and these changes were reversed slightly in the DOX+L group, although these differences were not significant (Figure 1(a)). Echocardiography and hemodynamic measurements demonstrated that DOX reduced cardiac systolic heart function, as demonstrated by decreased LVEF, LVFS, dp/dtmax, and dp/dtmin in DOX-treated mice. However, compared with the DOX group, the DOX+L group exhibited attenuated cardiac dysfunction, which was reflected by an improvement in LVEF, LVFS, dp/dtmax, and dp/dtmin (Figures 1(b)–1(d)). However, there was no difference in cardiac function between the CON and the CON+L groups.

### 3.2. Levosimendan Ameliorated the Myocardial Injury Induced by DOX

**Figure 2:**
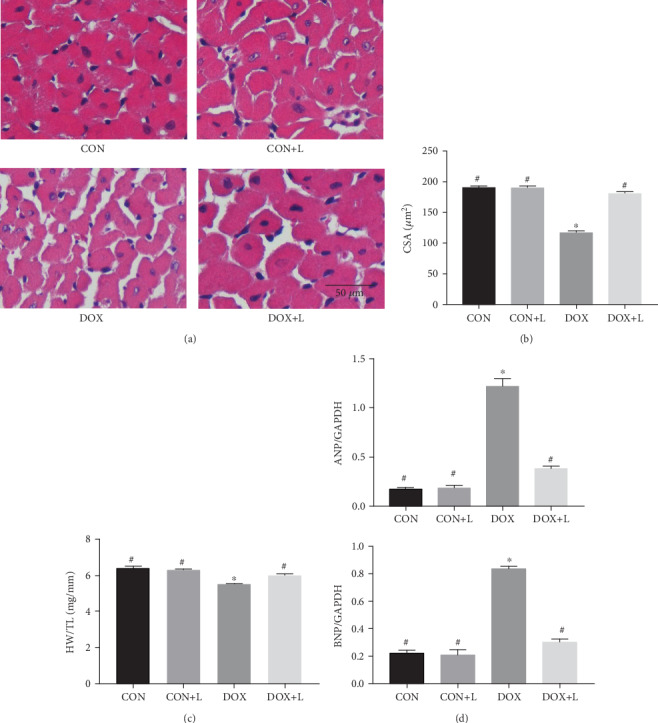
Levosimendan ameliorated the myocardial damage induced by DOX. (a) HE staining and representative images in mice. Scale bar: 50 *μ*m. (b) Statistical analysis of cross-sectional area (CSA, *n* = 100+ cells per experimental group). (c) The ratio of heart weight and tibia length (HW/TL) in each group. (d) Real-time PCR for the mRNA expression of cardiac injury-associated genes from the myocardium in each group, including ANP and BNP (normalized to GAPDH). ^∗^*P* < 0.05, vs. the CON group; ^#^*P* < 0.05, vs. the DOX group.

To observe the effect of levosimendan on the myocardial injury caused by DOX treatment, HE staining and real-time PCR were performed. The results of HE staining indicated that the reduced CSA of myocardial cells following DOX injection was restrained by levosimendan (Figures 2(a) and 2(b)). In addition, levosimendan could attenuate the body weight loss induced by DOX treatment. DOX injection resulted in a decreased HW/TL, yet levosimendan could improve HW/TL (Figure 2(c)). Moreover, as determined through PCR, indexes of cardiac injury, including the mRNA expression of atrial natriuretic peptide (ANP) and brain natriuretic peptide (BNP), were increased in the DOX group and were mitigated in the DOX+L group (Figure 2(d)).

### 3.3. Levosimendan Exhibited an Antiapoptotic Effect in DOX-Treated Mice

**Figure 3:**
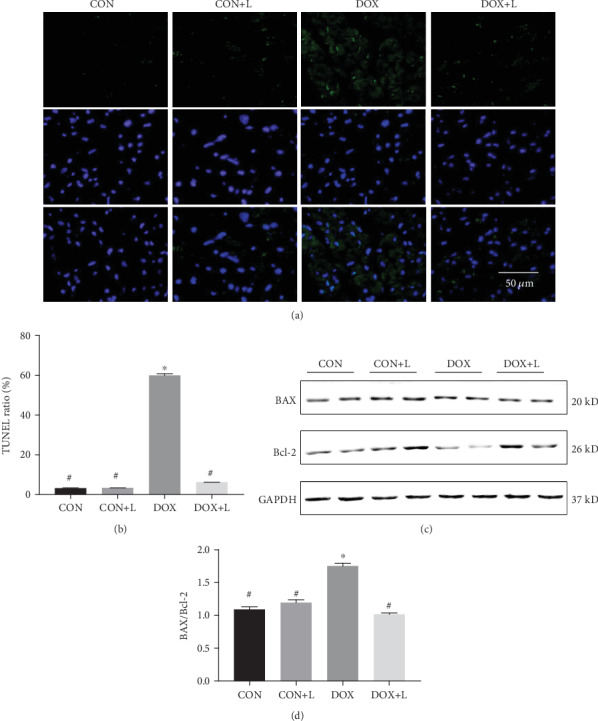
Levosimendan exhibited an antiapoptosis effect in DOX-treated mice. (a, b) Terminal deoxynucleotidyl transferase-mediated nick end labeling (TUNEL) assay for apoptosis in heart tissues and quantitation of the TUNEL ratio in the indicated group. Scale bar: 50 *μ*m. (c, d) Representative western blots of BAX, Bcl-2, and c-caspase-3, and quantitative analysis for BAX/Bcl-2 and c-caspase-3/GAPDH ratio. ^∗^*P* < 0.05, vs. the CON group; ^#^*P* < 0.05, vs. the DOX group.

To investigate the effects of levosimendan on myocardial apoptosis after DOX stimulation, TUNEL staining was performed. As shown in Figures 3(a) and 3(b), DOX injection resulted in a noteworthy increase in myocardial apoptosis, which was indicated by the increased number of TUNEL-positive cells, and levosimendan significantly restrained the apoptosis caused by DOX. Few TUNEL-positive cells were detected in the CON group and the CON+L group. We then measured the expression levels of apoptosis markers including BAX, Bcl-2, and c-caspase-3. We found that the injection of DOX resulted in increased proapoptotic BAX and c-caspase-3 and reduced antiapoptotic Bcl-2 expression compared with no DOX injection. Levosimendan treatment could reverse these alterations (Figures 3(c) and 3(d)). The expression of BAX, Bcl-2, and c-caspase-3 in the myocardium was not significantly altered in the CON and CON+L groups.

### 3.4. Levosimendan Inhibited Myocardial PTEN/Akt Signaling In Vivo

**Figure 4:**
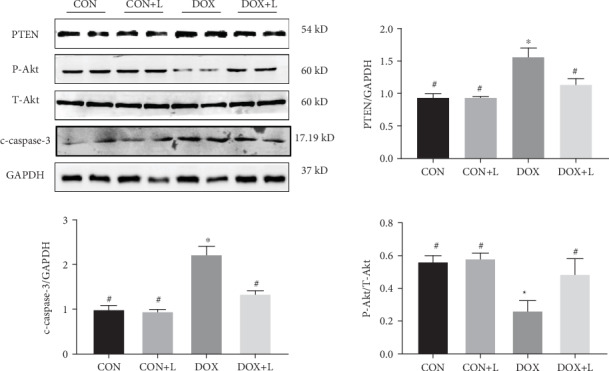
Levosimendan inhibited myocardial PTEN/Akt signaling in vivo. Western blotting and quantitative analysis of the PTEN/Akt pathway in cardiac tissue, including PTEN, P-Akt, and T-Akt protein level analysis. The ratio of PTEN/GAPDH and P-Akt/T-Akt was shown. ^∗^*P* < 0.05, vs. the CON group; ^#^*P* < 0.05, vs. the DOX group.

To examine the underlying mechanism of levosimendan, the PTEN/Akt signaling pathway was examined through western blotting. The results showed that DOX injection remarkably upregulated the expression of PTEN as well as significantly downregulating P-Akt expression. However, levosimendan appeared to reduce the level of PTEN and ameliorated the expression of P-Akt (Figure 4). These results suggest that levosimendan might protect against DOX-induced cardiotoxicity via inhibition of the PTEN/Akt pathway.

### 3.5. Levosimendan Suppressed DOX-Induced Cardiotoxicity via the PTEN/Akt Pathway In Vitro

**Figure 5:**
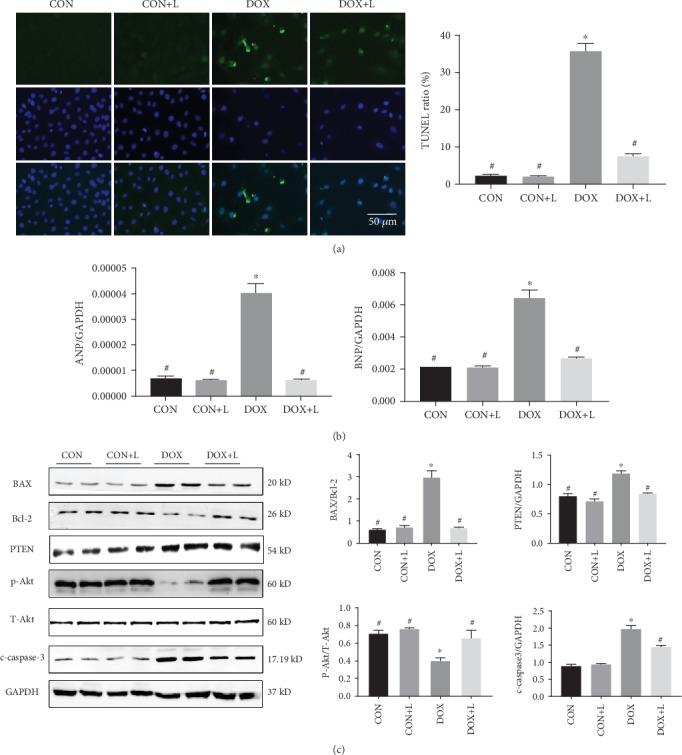
Levosimendan suppressed DOX-induced cardiotoxicity via the PTEN/Akt pathway in vitro. (a) TUNEL staining for apoptosis in H9C2 cells and quantitative analysis of the TUNEL ratio in each group. Scale bar: 50 *μ*m. (b) Real-time PCR for the mRNA expression of cardiac injury-associated genes in H9C2 cells in each group, including ANP and BNP (normalized to GAPDH). (c) Western blotting and quantitative analysis of apoptosis-related proteins and the PTEN/Akt signaling pathway in H9C2 cells, including BAX, Bcl-2, c-caspase-3, PTEN, P-Akt, and T-Akt. GAPDH was used as a loading control. ^∗^*P* < 0.05, vs. the CON group; ^#^*P* < 0.05, vs. the DOX group.

To verify the protective effect of levosimendan in DOX-induced cardiotoxicity and its molecular mechanism in vitro, cell experiments were performed. As shown in Figure 5(a), many TUNEL-positive cells were observed in H9C2 cells in the DOX group; in contrast, in the DOX+L group, TUNEL-positive cells were much less abundant (Figure 5(a)). In addition, western blot analyses revealed that the expression of proapoptotic BAX and c-caspase-3 was increased while that of antiapoptotic Bcl-2 was decreased in the DOX group compared with the CON group. Levosimendan could reverse these changes (Figure 5(c)). Levosimendan could also ameliorate DOX-induced cardiomyocyte injury, which was reflected by a decrease in ANP and BNP compared with the DOX group (Figure 5(b)). Furthermore, the PTEN/Akt signaling pathway, a key mechanism regulating the progression of cell growth and apoptosis, was inhibited by levosimendan in vitro.

### 3.6. Inhibition of Akt Abolished the Protective Effect of Levosimendan

**Figure 6:**
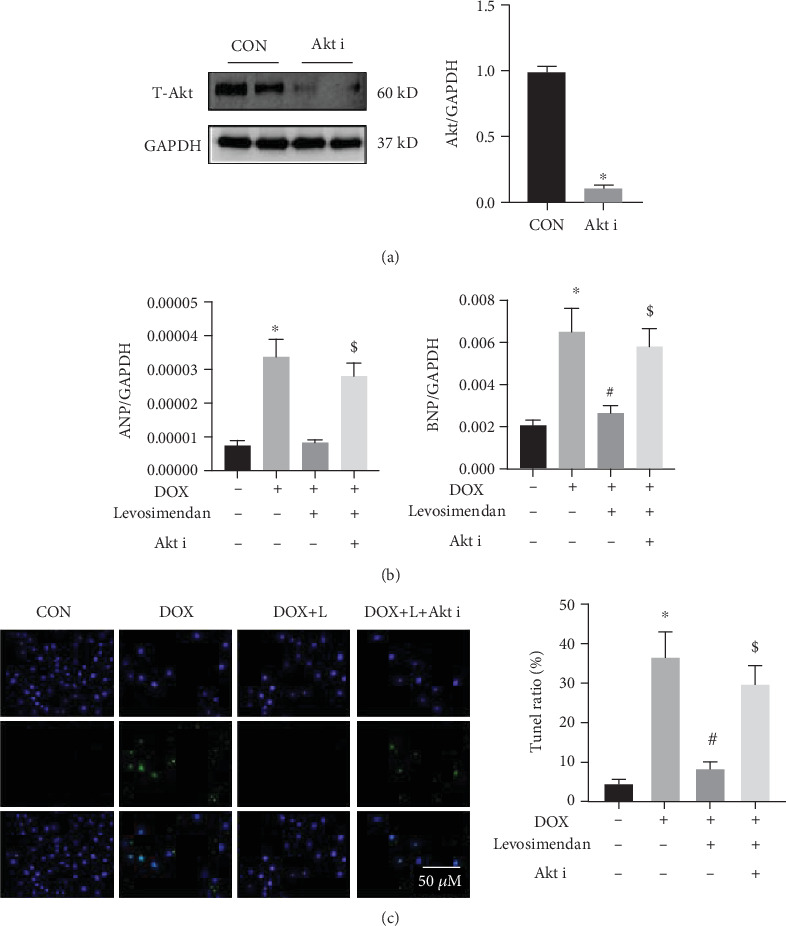
Inhibition of Akt abolished the protective effect of levosimendan in vitro. (a) Western blotting and quantitative analysis of Akt in H9C2 cells. (b) Real-time PCR for the mRNA expression of cardiac injury-associated genes in H9C2 cells in each group, including ANP and BNP (normalized to GAPDH). (c) TUNEL staining for apoptosis in H9C2 cells and quantitative analysis of the TUNEL ratio in each group. Scale bar: 50 *μ*m. ^∗^*P* < 0.05, vs. the CON group; ^#^*P* < 0.05, vs. the DOX group; ^$^*P* < 0.05, vs. the DOX+L group.

To investigate the PTEN/Akt signaling pathway in levosimendan-mediated protective effect during DOX, we pretreated H9C2 cells with the Akt inhibitor. As is shown in Figure 6(a), Akt inhibitor administration significantly decreased the expression of Akt in H9C2 cells. Then, we detected the cardiomyocyte injury and apoptosis. Expectedly, levosimendan evidently blocked H9C2 injury attributed by DOX; however, coadministration with Akt i eliminated the optimistic effect of levosimendan (Figure 6(b)). Additionally, the analyses of TUNEL indicated that levosimendan alleviated the apoptosis induced by DOX, nevertheless, Akt i removed the function of levosimendan (Figure 6(c)). These data suggested that Akt inhibition abolished the cardioprotection of levosimendan during DOX.

## 4. Discussion

In the current study, we demonstrated that levosimendan could attenuate DOX-induced cardiotoxicity by significantly protecting cardiac function and reducing myocardial injury and apoptosis in vivo and in vitro. Furthermore, we demonstrated that these protective effects were mediated by the inhibition of PTEN and the subsequent activation of Akt, both in vivo and in vitro. Herein, the current study provided a novel approach for the treatment of DOX-induced cardiotoxicity.

Apoptosis has been shown to be one of the key processes in DOX-induced cardiac injury [4, 8, 23, 24]. We observed few apoptotic cells in the CON and CON+L groups, but more apoptotic cells in the DOX group. Apoptosis, characterized by the orderly death of cells, was controlled by correlative genes, and apoptosis can be induced by many stimuli such as hypoxia, inflammation, and related drugs [23, 25]. Apoptosis is an active process that enables adaptation to external factors rather than a passive process, and excessive apoptosis is unfavorable. There are two relatively clear apoptotic pathways: the external pathway involves cleavage of the initiator procaspase-8 into the active caspase-8, thereby causing a series of apoptosis cascades once the Fas ligand binds to the Fas receptor on the cell surface [22, 26, 27]; and the endogenous pathway is strictly regulated by proteins from the Bcl-2 family and targets mitochondria, maintaining the competition between antiapoptotic Bcl-2-like survival factors and proapoptotic BAX-like death factors [22, 27, 28]. BAX and Bcl-2 belong to the family of Bcl-2 proteins. If BAX expression is increased, a homodimer of BAX/BAX is produced which promotes apoptosis; otherwise, a heterodimer or homodimer of Bcl-2/Bcl-2 is generated to inhabit apoptosis. The ratio of those two factors determines whether apoptosis occurs, as well as the severity of apoptosis. Furthermore, as the cytokines involved in the regulation of apoptosis, caspases are core participators in the activation of apoptotic cascades [16, 27, 28]. The concentration of active caspase-3 is closely associated with the rate of apoptosis. The current study showed that the expression of BAX and active caspase-3 proteins in the DOX group was upregulated dramatically both in vivo and in vitro and that the Bcl-2/BAX ratio decreased significantly. In addition, the expression of BAX and c-caspase-3 in the CON+L group was decreased.

DOX activates PTEN, thereby modulating the activity downstream of the PI3K/Akt pathway [27]. PTEN mutation is the most common mutation in tumor suppressor genes which may negatively regulate cell function [29–31]. It has been universally acknowledged that the most important role of PTEN is to regulate growth and development, including cell growth, adhesion, migration, invasion, and apoptosis, and it also serves as the key regulator of pathogenesis in a variety of cardiovascular diseases (CVD), probably via the PI3K/Akt signaling pathway [32–36]. Multiple physical and chemical stimuli can activate PI3K and thereby phosphorylate-Akt (p-Akt), which subsequently functions as a key factor in downstream effectors [37, 38]. Activated Akt may exert its antiapoptotic effect through the regulation of caspase-3. Caspase-3 is a vital effector of cell apoptosis, existing as a precursor under normal conditions, and it is activated during apoptosis. Procaspase-3 can be activated through cleavage, and activated caspase-3 is the most important terminal cleavage enzyme in apoptosis [39–41]. Early investigations suggested that the activation of Akt could effectively inhibit cardiomyocyte apoptosis and reduce the extent of myocardial injury. In the current study, we have shown that the PTEN/Akt pathway was activated in DOX-induced apoptosis.

Regarding levosimendan, previously reported experimental results have indicated that it could increase the sensitivity between Ca^2+^ and myofilaments by combining with myocardial troponin C to strengthen myocardial contraction without increasing the intracellular Ca^2+^ concentration and oxygen consumption in the myocardium [13, 16, 27]. Simultaneously, its vasodilatory and anti-ischemic effects might be mediated by the opening of adenosine triphosphate-sensitive potassium channels in vascular smooth muscle cells. Moreover, levosimendan may protect various organs, including the heart, kidney, lung, and liver, from apoptotic cell death, probably through the modulation of membrane potential, reactive oxygen species formation, and adenosine triphosphate-sensitive potassium channel activity, thereby regulating mitochondrial function [42–44]. Recently, it has been reported that levosimendan can significantly inhibit interleukin-1*β*-induced apoptosis in adult rat cardiac fibroblasts. This cytoprotective effect of levosimendan was suggested to be caused by the activation of Akt and the inhibition of inducible nitric oxide synthase (iNOS) expression and subsequent NO production [16]. In addition, a recent experiment showed that levosimendan pretreatment was associated with attenuated myocardial apoptosis and therefore partially reverses coronary microembolization-induced myocardial dysfunction in swine. The study also revealed that the potential mechanisms underlying the protective effects of levosimendan in coronary microembolization-induced cardiac dysfunction might involve the regulation of Akt [27].

In conclusion, the data in the current study demonstrate that levosimendan relieves DOX-induced myocardial injury and improves cardiac function. These protective effects involve regulation of the PTEN/Akt signaling pathway, which leads to reduced apoptosis. And levosimendan therapy targeting the PTEN/Akt pathway may be a promising therapeutic approach to treat chemotherapeutic agent-induced cardiotoxicity.

## Data Availability

Requests by researchers to access the data, analytic methods, and study materials for the purposes of reproducing the results or replicating procedures can be made to the corresponding author who manages the information or by contacting the corresponding authors (Deng, Wei (email: vivideng1982@whu.edu.cn) and Tang, Qi-Zhu (email: qztang@whu.edu.cn), address: Department of Cardiology, Renmin Hospital of Wuhan University, Jiefang Road 238, Wuhan 430060, China, Tel.: +86 27 88073385; fax: +86 27 88042292).
